# Supplementary material for: Radical pairs may explain reactive oxygen species-mediated effects of hypomagnetic field on neurogenesis
Source: PLoS Comput Biol. 2022 Jun 2;18(6):e1010198. doi: 10.1371/journal.pcbi.1010198 (PMC9197044; doi:10.1371/journal.pcbi.1010198)
Supplement: S1 Supporting information — (PDF) [file pcbi.1010198.s001.pdf]

S1 Supporting information: Effects of having additional nuclear spins on the triplet yield ratio (GMF to HMF) for a singlet-born radical pair

In the paper, we have considered only one HFI (with H5) for FADH$^\bullet$. In reality, the situation is more complex, and this radical interacts with multiple nuclear spins [1]. These additional interactions can have a significant effect on the spin dynamics of the radical pair. In this Supporting information, we have reproduced the Fig. 3 of the paper with one (N5 in Fig. A in S1 Supporting information) and two (N5 and N10 in Fig. B in S1 Supporting information) additional nuclear spins. The effect of this change is to reduce the maximum triplet yield ratio.

This might be thought to imply the need for lower relaxation rates. However, as pointed out in the paper, a small change at the level of ROS production does not necessarily mean a small change at the level of neurogenesis [2, 3]. Sies et al. [2] have mentioned that signaling by oxidants can be amplified by triggering kinase cascades. Much smaller alterations at the ROS level might result in the observed HMF effects. Therefore, such low relaxation rates of flavin radicals may not be required.

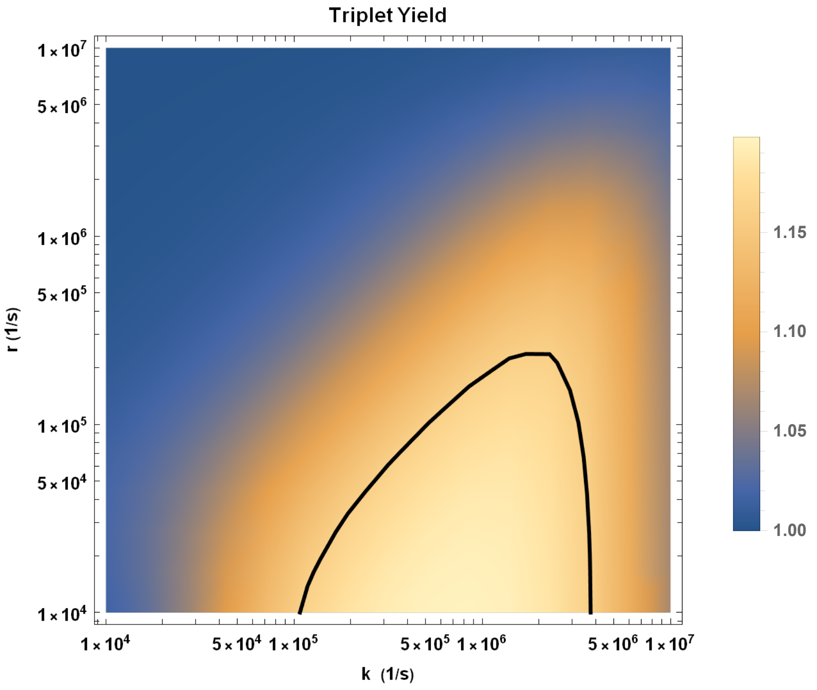

Fig A: **Triplet yield ratio (GMF to HMF) for singlet-born RP in the $k - r$ plane for two nuclear spins (H5 and N5).** The region below the solid black lines (1.16) is in agreement with the experimental range for the ratio of the numbers of BrdU+ cells after an 8 week exposure to GMF and HMF(1.34 ± 0.18) [4]. The value of HFCCs are $a_{H5} = -802.9\ \mu T$ and $a_{N5} = 431.3\ \mu T$ [1].

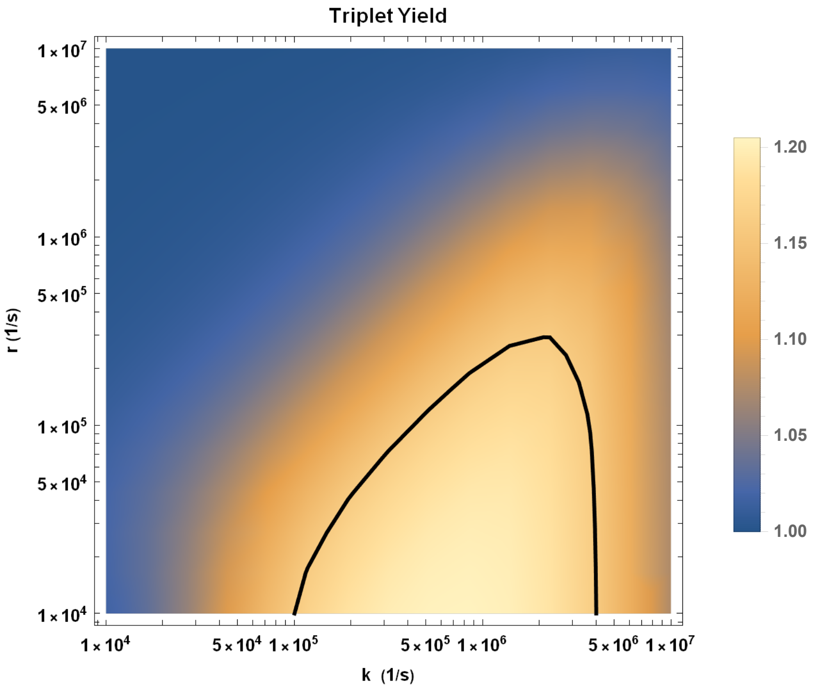

Fig B: **Triplet yield ratio (GMF to HMF) for singlet-born RP in the $k - r$ plane for three nuclear spins (H5, N5, and N10).** The region below the solid black lines (1.16) is in agreement with the experimental range for the ratio of the numbers of BrdU+ cells after an 8 week exposure to GMF and HMF(1.34 ± 0.18) [4]. The value of HFCCs are $a_{H5} = -802.9\ \mu T$, $a_{N5} = 431.3\ \mu T$, and $a_{N10} = 250.6\ \mu T$ [1].

References

[1] Lee AA, Lau JC, Hogben HJ, Biskup T, Kattnig DR, Hore P. Alternative radical pairs for cryptochrome-based magnetoreception. Journal of The Royal Society Interface. 2014;11(95):20131063.

[2] Sies H, Belousov VV, Chandel NS, Davies MJ, Jones DP, Mann GE, et al. Defining roles of specific reactive oxygen species (ROS) in cell biology and physiology. Nature Reviews Molecular Cell Biology. 2022; p. 1–17.

[3] Blume-Jensen P, Hunter T. Oncogenic kinase signalling. Nature. 2001;411(6835):355–365.

[4] Zhang B, Wang L, Zhan A, Wang M, Tian L, Guo W, et al. Long-term exposure to a hypomagnetic field attenuates adult hippocampal neurogenesis and cognition. Nature communications. 2021;12(1):1–17.
